# Surgery-First Approach for Dentofacial Deformity: A Systematic Review

**DOI:** 10.7759/cureus.35085

**Published:** 2023-02-16

**Authors:** Om Prakash, Santosh K Verma, Awanindra K Jha, Saurabh Mallick, Subia Ekram, Mukesh Soni

**Affiliations:** 1 Department of Oral and Maxillofacial Surgery, Dental College, Rajendra Institute of Medical Sciences, Ranchi, IND; 2 Department of Periodontology, Dental College, Rajendra Institute of Medical Sciences, Ranchi, IND; 3 Department of Orthodontic and Dentofacial Orthopedic, Dental College, Rajendra institute of medical sciences, Ranchi, IND; 4 Department of Oral and Maxillofacial Surgery, Mallick Dental and Maxillofacial Center, Ranchi, IND; 5 Department of Oral and Maxillofacial Surgery, Northern Railway Central Hospital, New Delhi, IND

**Keywords:** surgery-first approach, orthodontic, pico framework, orthognathic treatment, dentofacial

## Abstract

This review is based on the surgery-first approach for dentofacial deformity. This review has critically highlighted various promising aspects and factors associated with dentofacial deformity and can be viewed as valuable research work. In addition, this review highlights a systematic manner of surgery that can reduce the possible duration of treatment. The main findings of the review have established that the appropriate approaches to surgery can be beneficial for patients of any age group. The surgery-first approach is mainly utilized for tissue transfer as well as oral cancer as the first-line treatment. This critical review has successfully evaluated the limitations and advantageous traits of the specific surgery approach that has been outlined in this context. It has established the surgery approach as an effective measurement to reduce the time taken for treatment without compromising the patient’s health. In the final phase of this review, the accuracy and appropriateness of this surgery-first approach have been effectively demonstrated.

## Introduction and background

The surgery-first approach (SFA) refers to a stable and safe procedure undertaken for the rectification of dentofacial deformities. The adoption of the SFA in orthodontic or orthognathic surgery treatment may lead to the correction of facial deformities within a short amount of time. Therefore, the purpose of this study is to evaluate the safety of surgery as an approach to dentofacial deformity corrections by conducting a thorough systematic review. The study has also addressed the key problem related to this approach, relevant treatment or intervention measures, and their relevant outcomes for patients in accordance with the PICO framework. Following this framework, the study also conducted a systematic review of articles to critically evaluate the findings and limitations associated with the SFA in the existing literature.

The implementation of technology for careful interpretation, precise decision-making, and effective planning for the subsequent treatment measures is contributing to a better outcome. The comparison of age, gender, society, and environment has been done to understand the history behind the development of dentofacial deformities and evaluate their treatment procedures.

## Review

Methodology

Protocol and Eligibility Criteria for Information, Resources, and Search

To analyze the systematic review, the application of the PICO model is quite effective. For example, to conduct a systematic review of the effectiveness of the SFA, this study mainly investigated articles and journals that focus on patients with Class II and Class III “skeletal deformities” as the “problem” areas. In addition, the included articles have been written in English and can be downloaded in PDF format. Keywords such as “dentofacial deformity,” “surgery-first approach,” “SFA,” and “orthognathic treatment” were used to search databases for information and resources.

Study Selection, Data Items, and Data Collection

**Figure 1:**
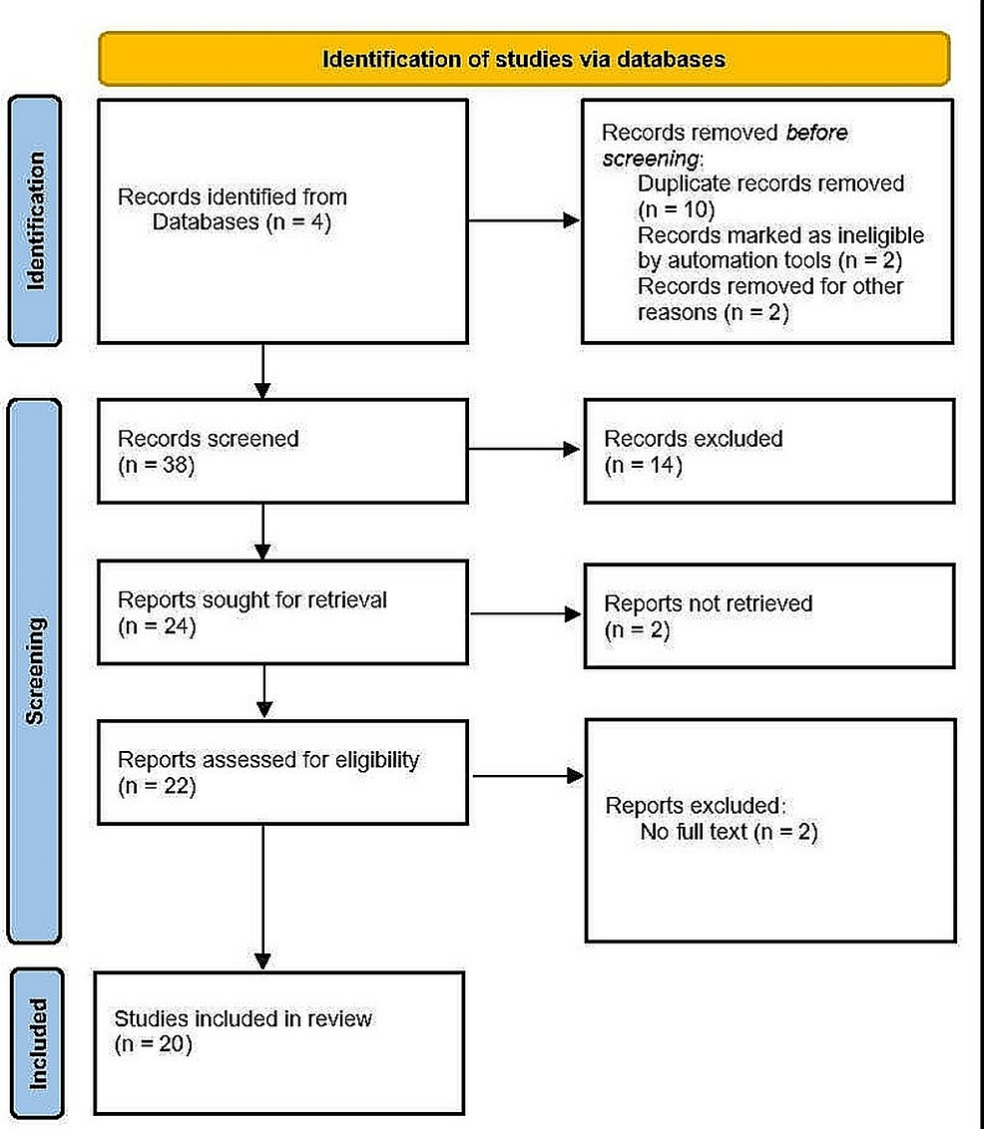
PRISMA framework protocol.

In this study, 20 scholarly articles and academic journals were selected for systematic review. The resources were collected based on relevant keywords and their reliability level. Articles that were published in the last five years were included. In addition, these resources have been collected from authentic databases such as ProQuest, ResearchGate, ScienceDirect, and PubMed. For the selection of articles and journals, the PRISMA framework was applied to ensure precision (Figure 1).

Results

General Characteristics

In this study, 20 scholarly articles were included. Among these articles, four have focused on analysis for developing the discussion; the remaining 15 have been developed using primary qualitative or quantitative data. However, in this systematic review, there was also an article that implemented a mixed-methods approach, generating both primary and secondary findings. Among the 15 primary studies, three were cohort studies and three were observational studies. The rest of the primary studies were mostly case studies (Table 1).

**Table 1:** List of articles included for review.

Article	Author name and Year	Outcomes (Out of 5)
Quality of life changes in Taiwanese dentofacial deformity patients undergoing orthognathic surgery	Ng et al. 2022 [1]	3
The ortho-surgical procedure involving mandible, maxilla, mento and associations: a systematic review	Avendaño et al 2022 [2]	3.7
TMJ position in symmetric dentofacial deformity	Ravelo et al 2022 [3]	2.7
Surgical occlusion setup in correction of skeletal class iii deformity using surgery- first approach: guidelines, characteristics and accuracy	Liao and Lo, 2018 [4]	3.5
The current concept of the surgery-first orthognathic approach	Choi and Lee, 2021 [5]	4
Surgery first: current state-of-the-art orthognathic surgery and its potential as a primary treatment modality in obstructive sleep apnea with concurrent dentofacial deformities	Pearl et al. 2022 [6]	3
Surgery-first approach using virtual setup for the treatment of dentoskeletal deformities	Pradeepthi et al. 2018 [7]	2.2
Modern surgery-first approach concept in cleft-orthognathic surgery: a comparative cohort study with 3D quantitative analysis of surgical-occlusion setup	Seo et al. 2019 [8]	4.5
Influence of personality traits on a patient’s decision to accept orthognathic surgery for correction of dentofacial deformity	Vidakovica et al. 2022 [9]	3
Dentofacial deformity: treated with combined orthodontic and orthognathic surgery treatment	Althagafi and Korayem, 2020 [10]	3.5
Quality of life measurement following a double jaw correction for dentofacial deformities: a single-centre retrospective analysis	Serhat et al. 2021 [11]	3
Framework for improved surgical-orthodontic collaboration in care and management of patients with dentofacial deformity	Dos Santos et al. 2020 [12]	3.6
Complex approach to surgical treatment of external nose deformations in combination with jaw-dental anomalies	Khalmatova et al. 2021 [13]	4
Surgery-first approach using virtual setup for the treatment of dentoskeletal deformities	Qadry et al. 2021 [14]	4
Oral health-related quality of life changes in patients with dentofacial deformities Class II and III after orthognathic surgery: a systematic review and meta-analysis	Duarte et al. 2022 [15]	4.5
Surgery first approach in the management of mandibular prognathism coexisting with maxillary fibrous dysplasia: A case report	Agarwal et al. 2019 [16]	4
Concepts, protocol, variations and current trends in surgery first orthognathic approach: a literature review	Mahmood et al. 2018 [17]	2.8
The impact of a surgery-first approach on oral health-related quality of life	Vongkamolchoon et al.2021 [18]	2.5
Surgery first - an expedited correction of dentofacial deformity	Singh et al. 2021 [19]	2.5
Skeletal stability in orthognathic surgery with the surgery first approach: a systematic review	Soverina et al. 2019 [20]	3.5

Synthesis of the results

The results of the selected studies, although diverse in nature, mostly focus on analyzing the importance of the SFA approach in mitigating various dentofacial deformities. For example, the cohort study by Ng et al. [1] has been found to be largely related to the “skeletal class III” patients. In their sample, 84.4% of the patients were found to benefit from surgery. Additionally, symmetric and asymmetric patients who received the SFA benefitted from gaining self-motivation and confidence. Further, in the study by Avendao et al. [2], of the 107 articles associated with the relevant subjects, individuals with “class III dentofacial deformities” have been addressed in this research. Observations through the coronal section have defined that the mediolateral distance is greater in class III than in class II after conducting a statistical analysis [3]. As per the findings of Agarwal and Khan [16], the primary aim of this treatment is the achievement of symmetry within the facial profile as well as the conservative management of the lesion, as osteotomies along with “dysplastic jaw bones” have created a significant challenge for fixation only through treatment. As a result, orthognathic surgery has been performed before orthodontic treatment under transnasal intubation and general anesthesia. According to a primary finding in Liao and Lo [4], the implementation of the SFA for these patients helped improve the symmetry of their jawlines. As a result, patients who underwent surgery required no additional occlusal set-up for treating dentofacial deformities.

In contrast, according to the secondary findings of Choi and Lee [5], the advantage of the SFA lies in its ability to possibly reduce treatment time and its patient-orientedness. However, it should also be noted that the difference between “cephalometric landmarks” in both mandibular and palatal angles has created certain controversies regarding the stability of the SFA. However, a significant finding was obtained in the observational study by Pearl et al. [6] using primary and secondary data. According to the findings of their study, the application of the SFA evidently reduced treatment time for individuals suffering from dentofacial deformities. In addition, this method has also been equally significant for decreasing distortion within anterior-posterior facial dimensions, malocclusions, and the shape of the jawline.

The outcomes of another observational study by Pradeepthi et al. [7] demonstrated that the SFA for planning virtual surgeries in orthodontic treatment requires careful supervision. The implementation of 3D planning enhances the preoperative diagnosis and the decision-making process for the surgery as well. In addition, the application of cutting guides and occlusal splints allowed for transforming virtual planning into reality in the SFA in this study. Another study to focus on 3D planning was the study by Seo et al. [8]. As per the results of this study, no additional occlusal setup was required to develop the virtual planning for the SFA. In addition, this approach also reduced the risks of developing wounds, infections, or hemorrhages.

Discussion

From the findings of this systematic review, it was observed that, in cases of medical complications, it becomes essential to undertake an SFA for individuals with class II and class III skeletal deformities. The application of this approach becomes effective in reducing the time for treatment and the effort for further supervision of this complication. Additionally, the findings of the research showed that patients who have lower self-esteem and are older have taken up the challenge. They are found to be concerned with facial esthetics, social aspects, implementation of oral, and impairment associated with oral functions. Dentofacial deformities can occur among patients of different age groups. However, the patient’s confidence level can alter. For this reason, some of the patients accepted the treatment, while others did not have self-esteem issues or perfectionism concerns.

Strengths and Limitations

This systematic review has certain strengths and limitations. Concerning strengths, the studies included in this systematic review have mostly been primary observational studies, cohort studies, and case studies. As a result, this study focused mostly on evidence-based data on the importance of the SFA in the cases of patients who had a medical situation that required alignment, increased leveling, or sensitive extractions but were unable to undertake this approach due to its pre-operative nature. Hence, such studies further enhanced the importance of the SFA in medical scenarios and increased the accuracy and reliability of the findings. However, concerning limitations, certain articles implemented a general approach toward the SFA approach and its application in dentofacial treatment. Thus, some parts of the findings lacked specificity due to the inclusion of secondary studies. Similarly, it should also be noted that this study also lacked sufficient generalization of its outcomes, as the nature of the findings in the selected outcomes appeared to be entirely different.

Implications for Clinical Findings

The findings of this study have primarily focused on evaluating the importance of the SFA in the treatment of dentofacial deformities, especially in patients with class II and class III anomalies in the jawline, face, and dental orientation. For instance, Vidakovic et al. [9] aimed to illustrate the role of orthognathic surgery in increasing the quality of life of patients aged 14-53 years and understand the influences on their personality traits based on the decision to accept surgery. A total of 108 patients aged between 14 and 53 years were included in this study; these patients received orthognathic functional treatment to correct the dentofacial deformity. Personality traits were considered by examining extraversion, neuroticism, agreeableness, openness, consciousness, perfectionism, self-esteem, dimensions, and the orthodontic quality of life questionnaire. The responses of patients who accepted orthodontic surgery and those who refused to it were compared. The study findings showed that patients with lower self-esteem and those who are older have taken up the challenge. These patients were found to be concerned with facial esthetics, social aspects, implementation of oral, and impairments associated with oral functions. The dentofacial deformity can occur among patients of different age groups. Nonetheless, the patient’s confidence level can alter. For this reason, some of the patients accepted the treatment, while others did not have self-esteem issues or perfectionism concerns. However, the study was limited to personality traits rather than patients’ complex medical issues and the reasons for dentofacial deformity development.

Further, Althagafi and Korayem [10] aimed to analyze recently developed treatment processes, including orthodontic and orthognathic treatment, and illustrate the anticipated benefits of understanding the issues related to class II and class III dentofacial deformities. To illustrate the phases, certain case studies have been taken into consideration to outline class III malformations. In this regard, a systematic analysis of the issues and probable solutions associated with jaw surgery was addressed. It was observed that the orthognathic process to overlay the pre-operative circumstances is an effective option to treat dentofacial deformity. However, this study is limited due to its focus on ideal adjunct oral photographs, psychosocial well-being, and a multidisciplinary team approach and the exclusion of complex medical issues in different age groups and regions from the research.

Dundar et al. [11] aimed to investigate the perioperative process with the help of cephalometric measurements in order to understand the objectives behind and measurement of quality of life using double jaw surgery. Thus, the methods in this study followed the preoperative process to treat dentofacial deformities with double surgery. The motives behind the surgical treatment and the post-surgery patient satisfaction questionnaire were included to understand the degree of post-treatment satisfaction of patients. It was observed in this study that the patients confirmed their satisfaction with the operational process and reported an increase in self-confidence. Functional and aesthetic motivations are the core values of the treatment depicted in the research process. However, as for limitations, it can be stated that the high level of complexity associated with orthognathic surgery using the perioperative process was not critically analyzed in this research. The lack of quantifiable data also hindered the standard parameters of research.

According to Santos et al. [12], the resources from the collaborative management of the patients from Nigeria were the most affected, and the care protocols associated with surgical orthodontic care, surgical orthodontics, orthognathic surgery, alternative orthognathic surgery, and post-traumatic facial deformity were determined as the most important parameters. Since the article aimed at understanding the effectiveness of collaboration between the Department of Oral Maxillofacial Surgery and Obafemi Awolowo University Teaching Hospital to critically justify the collaboration of dentofacial deformity, maxillofacial surgeons, and orthodontists, the methods of desk review and the relevant publications and existing practices with guidelines related to dentofacial deformity treatment were addressed. Using the data collected from the monthly meetings held by the departments of oral and maxillofacial surgery and orthodontic units, the factors associated with the treatment of surgical orthodontic care were illustrated. However, the limitations of this research were related to the critical understanding of the social values of the treatment as a collaboration among different treatment departments was being addressed, although the care conception and proper management methods for sustaining mental satisfaction and boosting the confidence level of patients were lacking.

Khalmatova and Normurodova [13] aimed at determining the complexities and challenges of facial deformities and their relations with jaw dental anomalies. Hence, to collect information on the relevant aspect, 100 participants from different age groups with prevalent deformities were taken into consideration. Their study primarily addressed people with deformity in the nose (70%), while the remaining patients (30%) had dentoalveolar complex issues. The findings suggest that the extensive manifestation of the critical issues and examinations using X-ray, endoscopy, and other critical factors of the treatment process of dental deformities were defined in this research. However, this study is limited to the generalised values of medical treatment for dental deformities, and information regarding critical medical complexities, post-medical conditions, and others was not offered in this research.

Qadry et al. [14] aimed at emphasizing virtual platforms such as computer-assisted surgical simulations for the SFA in the cases of endoskeletal deformities. In their study, 10 participants with dentofacial deformities were considered for data collection based on subjective parameters. The tools used in the phases include evaluation, photographs, study materials, 2D lateral cephalometry, optical scanning, and others. The results evaluated indicated that there is no significant difference between the outcomes obtained from the planned and prospective 3D cephalometric analysis. As for the study limitations, virtual surgical planning and design include a lot more accuracy, while the present transfer of data and treatment process was dependent on digital tools. A minor error in the analysis and calculation can affect the outcome of the treatment.

In addition, the study by Duarte et al. [15] is also significant, as this study aimed to understand the combined effects of orthodontic and surgical treatments with critical justification on the oral health-related quality of life (OHRQoL). Hence, the study was done by searching databases such as PubMed, Embase or MEDLINE, Scopus, and Cochrane. The outcomes of orthodontal surgery and case II and case III category evaluations were critically addressed in this research through sensitivity analysis. As per the findings, the global scores obtained from the SMD and OHRQL improvements of the patients on a psychological and physical level were classified in this research. Through sensitivity analysis, it was found that functional limitations were lower among class II patients. This study was limited in its consideration of data related to material methods, while the critical emphasis on medical processes and therapeutic instruments was not properly addressed.

Ng et al. [1] aimed to investigate the role of the environment, living standards, and practices in orofacial deformity. To this end, the research focused on 113 participants from Taiwan who experienced the critical complexities of orthognathic surgery. Standardized questionnaires with subgroup comparisons between class II- and class III-category patients with dentofacial deformity were implemented in this research. Orthognathic surgery guarantees a positive outcome on the generic health, aesthetics, and quality of life of dentofacial deformity patients, although there is an absolve of prospective evasion of the contemporaneous questionnaire. In contrast, Avendao et al. [2] conducted a critical study with the help of qualitative and quantitative data, and PRISMA analysis was implemented in this study. The research objective was to find surgical procedures to treat deformities by orthognathic surgery on the bones (maxilla and mandible). However, the research was limited to surgical outcomes on different bones, while the effective treatment methods and medical terminologies with illustrations of the surgical procedures were not included.

The research conducted by Ravelo et al. [3] aimed at understanding the role of “facial class,” the presence of malocclusion, and the mandibular plane to relate them with condyle position for the evaluation of their roles in symmetric dentofacial deformity. Hence, the classification of different age categories, gender deference, and anomalies on the skeletal class and mandibular plane were illustrated with qualitative and quantitative data. The subjective information was limited to medical treatments, while the social value and psychological impact of deformation in teeth and other parts of the skull on the patients were not addressed properly.

On the other hand, Agarwal and Khan [16] aimed to evaluate single-stage surgical correction for treating gnathic deformities caused by maxillofacial fibrous dysplasia and dentofacial deformities. To achieve this objective, an observational study was conducted on an 18-year-old female patient with asymmetrical facial composition and a displaced lower jaw. However, the results of the study were specific and lacked generalizability. The study by Liao and Lo [4] developed primary findings. Their study aimed to develop guidelines for surgical occlusion in association with the SFA in orthognathic treatment. To conduct the study, the researchers adopted a primary observational design with 53 skeletal class III patients with a bilateral sagittal osteotomy having a split. However, the primary observational design of the study lacked enough theoretical support from the existing literature.

Choi and Lee [5] aimed to critically analyze the contemporary concept of the SFA in orthognathic treatment to deal with dentofacial deformities. To conduct their study, the researchers adopted a secondary thematic analysis over the available qualitative data in the existing literature. The secondary qualitative findings lacked the reliability of primary observations. The secondary study conducted by Mahmood et al. [17] aimed at critically evaluating the protocols adopted in the SFA for treating dentofacial deformities and analyzing the variations in their implementation. The SFA has been effective for the rectification of dental, facial, and jaw-related deformities. The standard protocol for applying the SFA can be classified into the preoperative stage, surgical stage, and postoperative stage. However, variations were also found in terms of computer-aided surgical stimulation and splint fabrication processes. This secondary study lacked the accountability of primary findings and the reliability of quantitative data.

In contrast, an observational mixed-methods study was conducted by Pearl et al. [6] to identify the current art of conducting surgery with the SFA in orthodontic treatment and its role in defying obstructive sleep apnea in association with concurrent dentofacial deformities. To mitigate the research purpose, this study derived its findings based on two observational case studies and a theoretical discussion. As a result, this study adopted a mixed method for research through a primary observational study and a secondary thematic analysis. Despite the mixed-methods approach, since the study was primarily based on qualitative data, it lacked the accuracy of quantitative data.

Pradeepthi et al. [7] aimed to measure the accuracy of the SFA in planning virtual surgeries in orthognathic studies. To meet the research aim, the study conducted a primary observational study and quantitative statistical analysis of the data derived from the preoperative and clinical evaluation of the patients, photographs, and the 2D images of lateral cephalometry. In addition, the study also analyzed the findings using DICOM and STL formats and used MIMICS software to develop a composite 3D model. Our review majorly focused on the study’s primary findings; hence, the study lacked enough emphasis on the theoretical discussion of the advantages and disadvantages of virtual surgery planning.

Seo et al. [8] offered a finding on the application of 3D models in their cohort study. The study focused on critically evaluating the 3D characteristics of surgical occlusion setup in mitigating dentofacial deformities caused by cleft formation. To conduct their study, the researchers focused on developing a primary cohort study based on the 3D images of 44 cleft cohorts with class III skeletal deformity and the datasets of 22 non-cleft cohorts with no dentofacial deformity. However, their study lacks reliability due to the absence of “inter-investigation” through quantitative analysis, and their findings have been derived from a limited number of individuals.

Vongkamolchoon et al. [18] conducted a cohort study with over 120 participants with class III skeletal asymmetry and bimaxillary protrusion. According to their findings, with the efficient implementation of the SFA, improvements were observed in patients with bimaxillary protrusion as well as those with class III skeletal asymmetry.

In contrast to the above studies, Singh et al. [19] focused on a secondary approach. Their study aimed to evaluate the conventional protocols for developing an SFA for dealing with dentofacial deformities and their disadvantages. To meet the research aims and objectives, the study conducted a secondary thematic analysis of the qualitative resources available within the existing literature. As per the findings of the following study, the SFA is crucial for correcting dentofacial disorders and treating malocclusions, as alveolar osteotomies help reduce resistance to the movement of teeth. However, it also has certain disadvantages, such as the SFA may fail to treat multiple disorders in a single application. The study has been solely based on secondary findings. Hence, it lacks the reliability of primary quantitative data.

Another significant cohort study was the study by Vongkamolchoon et al. [18]. Their study aimed to measure the longitudinal alterations that occur in dentofacial structure with the application of the SFA in association with the age, gender, and facial types of the individuals. The prospective cohort study included over 120 participants with class III skeletal asymmetry and bimaxillary protrusion. This study focused only on the longitudinal analysis of the participants and analyzed the findings only with the generalized estimating equations model.

Last but not the least, the secondary thematic study conducted by Soverina et al. [20] implementing the PRISMA framework suggested that the application of the penultimate time frames in both post-surgical and after-debonding periods has increased the stability of results derived from the SFA. Their study aimed to evaluate the probable skeletal relapse during the application of the SFA in treating dentofacial abnormalities.

As for limitations, the journals and articles collected for this review are case studies focused primarily on patients with class III skeletal deformities, with only one article focusing on patients with class II deformities.

## Conclusions

The SFA for dentofacial deformity is a systemic approach that can cure a patient within a short period of time without causing any health problems. The entire procedure is carried out in a methodical and meticulous manner. This approach is a very helpful way of treating dentofacial deformities. This systemic approach has some advantages that can be very beneficial to patients, although disadvantages exist. In this review, we discussed some disadvantages because of the short duration of the treatment. However, in most cases, the SFA is the most effective and systematic method of treating dentofacial deformities.
